# Supplementary material for: Proteomics signature of moderate-to-vigorous physical activity and risk of multimorbidity of cancer and cardiometabolic diseases
Source: Commun Med (Lond). 2026 Mar 13;6:160. doi: 10.1038/s43856-026-01514-9 (PMC13009281; doi:10.1038/s43856-026-01514-9)
Supplement: Supplementary file 2 — Supplementary Material [file 43856_2026_1514_MOESM2_ESM.pdf]

Proteomics signature of moderate-to-vigorous physical activity and risk of multimorbidity of cancer and cardiometabolic diseases

Supplementary material

Content

Supplementary Table 1. ICD codes for cancer, cardiovascular disease, and type 2 diabetes

Supplementary Table 2. Case numbers for each transition

Supplementary Table 3. Proteins associated with diseases as well as multimorbidity

Supplementary Table 4. Model performance metrics for single and mutually adjusted models for the proteomics signature score and physical activity

Supplementary Table 5. Hazard ratios and 95% confidence intervals for the proteomic signature and physical activity under single and mutual adjustment scenarios

Supplementary Figure 1. Flowchart of participant inclusion

Supplementary Figure 2. Continuous hazard ratios for the top hits among all proteins for the disease-free to incident disease transitions

Supplementary Figure 3. Continuous hazard ratios for the top hits among all proteins for the incident disease to subsequent multimorbidity transitions

Corresponding author: Michael J. Stein, Tel.: +49 941 944 521 6, Mail: michael.stein@ur.de, Department of Epidemiology and Preventive Medicine, University of Regensburg, Regensburg, Germany

Supplementary Table 1. ICD codes for cancer, cardiovascular disease, and type 2 diabetes

**Cancer**	**ICD-10 codes**	**ICD-O-3 codes**
Esophagus (adeno)	C15	8140, 8141, 8143–8145, 8190–8231, 8260–8263, 8310, 8401, 8480–8490, 8550–8551, 8570–8574, 8576
Stomach (cardia)	C16.0	8140–8145, 8147, 8210, 8211, 8214, 8220, 8221, 8230, 8231, 8255, 8260–8263, 8310, 8480, 8481, 8490, 8510, 8560, 8562, 8570–8576
Colorectum	C18–C20	
Breast	C50	
Corpus uteri	C54.0, C54.1, C54.2, C54.3, C54.9, C55	
Kidney (renal cell)	C64	8050, 8140, 8260, 8270, 8280–8312, 8316–8320, 8340–8344
Bladder	C67	
**Cardiovascular disease**		
Angina pectoris	I20	
Acute myocardial infarction	I21	
Other acute ischemic heart diseases	I24	
Chronic ischemic heart diseases	I25	
Atrial fibrillation	I48	
Other cardiac arrhythmias	I49	
Heart failure:	I50	
Cerebrovascular diseases (incl. stroke)	I60 to I69	
Atherosclerosis	I70	
Other peripheral vascular diseases	I73	
**Type 2 diabetes**	E11	
ICD: international classification of diseases, SCC: squamous cell carcinoma		

Supplementary Table 2. Case numbers for each transition

	From				
	**Baseline**	**Cancer**	**CVD**	**T2D**	**Sum**
**To**					
Baseline	27360				27360
Death	530	146	334	43	1053
Cancer	1108	772			1880
CVD	3445		2909		6354
T2D	1363			898	2261
Cancer-T2D		48			48
Cancer-CVD		142			142
CVD-Cancer			79		79
CVD-T2D			123		123
T2D-Cancer				41	41
T2D-CVD				381	381
CVD: cardiovascular disease; T2D: type 2 diabetes.					

Supplementary Table 3. Proteins associated with diseases as well as multimorbidity

Protein	Transition	HR (95% CI)	q	P-nonlinear
**Inversely associated with physical activity**				
GGT1	Baseline to CVD	1.07 (1.03, 1.12)	1.60E-02	4.30E-01
	Baseline to T2D	2.14 (1.84, 2.49)	7.80E-51	4.80E-05
	CVD to Multimorbidity	1.32 (1.12, 1.57)	1.50E-02	6.40E-01
**Positively associated with physical activity**				
ALPP	Baseline to CVD	1.04 (0.96, 1.13)	9.30E-05	1.10E-04
	Baseline to T2D	1.18 (1.10, 1.26)	1.10E-05	5.30E-01
	CVD to Multimorbidity	2.05 (1.34, 3.12)	2.50E-02	4.10E-03
CA14	Baseline to CVD	0.92 (0.84, 1.01)	3.10E-04	1.30E-02
	Baseline to T2D	0.53 (0.45, 0.61)	4.90E-32	7.30E-04
	Cancer to Multimorbidity	0.64 (0.47, 0.87)	2.20E-02	5.70E-01
CD34	Baseline to CVD	0.90 (0.86, 0.94)	3.00E-05	5.80E-01
	Baseline to T2D	0.69 (0.60, 0.80)	5.90E-10	1.90E-02
	CVD to Multimorbidity	0.65 (0.47, 0.88)	2.90E-02	5.80E-01
CD99L2	Baseline to CVD	1.05 (0.96, 1.14)	6.60E-03	1.40E-02
	Baseline to T2D	0.88 (0.82, 0.94)	2.30E-03	8.50E-02
	T2D to Multimorbidity	1.42 (1.14, 1.76)	1.00E-02	3.20E-01
CDCP1	Baseline to CVD	1.15 (1.10, 1.20)	8.80E-08	9.50E-02
	Baseline to T2D	2.19 (1.88, 2.55)	1.20E-50	5.50E-06
	CVD to Multimorbidity	1.32 (1.09, 1.60)	2.60E-02	1.40E-01
CHRDL2	Baseline to Cancer	1.18 (1.10, 1.27)	8.20E-05	7.60E-01
	Baseline to T2D	1.22 (1.14, 1.31)	4.10E-07	6.30E-01
	CVD to Multimorbidity	1.35 (1.10, 1.67)	2.60E-02	2.80E-01
IGFBP1	Baseline to CVD	1.08 (1.03, 1.14)	1.70E-02	6.40E-01
	Baseline to T2D	0.52 (0.47, 0.56)	1.20E-50	4.10E-01
	T2D to Multimorbidity	1.37 (1.09, 1.73)	3.60E-02	3.90E-01
LILRA5	Baseline to CVD	1.10 (1.05, 1.15)	5.00E-04	2.00E-01
	Baseline to T2D	1.31 (1.22, 1.41)	1.70E-11	1.40E-01
	Cancer to Multimorbidity	1.53 (1.11, 2.13)	4.90E-02	9.50E-01

CI: confidence interval; CVD: cardiovascular disease; HR: hazard ratio; T2D: type 2 diabetes.
Cox proportional hazards models were used, two-sided, with P-values obtained from two-sided likelihood ratio tests. q-Values are P-values adjusted for multiple testing using the false discovery rate method.
All models were stratified by age at baseline (5-year increments), sex, and country (England, Scotland, Wales), and adjusted for education level (highest, intermediate, lowest, none of those), socio-economic status (Townsend index, categorized using tertiles, missing values coded as missing), smoking (never, former, current), alcohol use (never, former, current), sedentary behavior (0-3h, 4-5h, 6-7h, >8h of daily TV watching, PC use during leisure, and driving), and screening for breast and/or bowel cancer (binary) as categorical variables, as well as physical activity (MET-hours), body mass index (kg/m2), and diet (healthy diet score, 0-7 scale) as continuous variables.

Supplementary Table 4. Model performance metrics for single and mutually adjusted models for the proteomics signature score and physical activity

Outcome	Base model	Model with signature score	Model with MVPA	Model with signature score + MVPA
**Baseline to cancer**				
AIC	16,007.5	15,996.5	15,998.6	15,993.2
LRT X^2	44.4	56.9	55.2	62.3
Overall P-value	6.92×10^{-3}	2.72×10^{-4}	4.62×10^{-4}	8.12×10^{-5}
C-index	0.715	0.712	0.714	0.713
**Baseline to CVD**				
AIC	49,489.9	49,490.6	49,491.4	49,492.4
LRT X^2	264.3	265.6	264.8	265.8
Overall P-value	$<1.00\times10^{-30}$	$<1.00\times10^{-30}$	$<1.00\times10^{-30}$	$<1.00\times10^{-30}$
C-index	0.658	0.658	0.658	0.658
**Baseline to T2D**				
AIC	18,695.4	18,610.6	18,686.2	18,612.4
LRT X^2	1,238.5	1,325.3	1,249.7	1,325.5
Overall P-value	$<1.00\times10^{-30}$	$<1.00\times10^{-30}$	$<1.00\times10^{-30}$	$<1.00\times10^{-30}$
C-index	0.805	0.812	0.805	0.812
**Cancer to multimorbidity**				
AIC	680.1	681.9	682.0	683.7
LRT X^2	23.3	23.5	23.4	23.8
Overall P-value	4.42×10^{-1}	4.88×10^{-1}	4.95×10^{-1}	5.33×10^{-1}
C-index	0.760	0.760	0.760	0.762
**CVD to multimorbidity**				
AIC	1,429.0	1,426.7	1,430.3	1,428.7
LRT X^2	74.1	78.3	74.8	78.4
Overall P-value	2.81×10^{-7}	1.11×10^{-7}	4.03×10^{-7}	2.04×10^{-7}
C-index	0.795	0.796	0.797	0.796
**T2D to multimorbidity**				
AIC	1,287.7	1,286.6	1,289.7	1,288.6
LRT X^2	19.4	26.5	19.4	26.5
Overall P-value	6.77×10^{-1}	4.36×10^{-1}	7.30×10^{-1}	4.89×10^{-1}
C-index	0.675	0.690	0.675	0.690

AIC: Akaike information criterion; CVD: cardiovascular disease; LRT: log-likelihood ratio test; MVPA: moderate-to-vigorous physical activity; T2D: type 2 diabetes.

Cox proportional hazards models were used, two-sided, with P-values obtained from two-sided likelihood ratio tests.

All models were stratified by age at baseline (5-year increments), sex, and country (England, Scotland, Wales), and adjusted for education level (highest, intermediate, lowest, none of those), socio-economic status (Townsend index, categorized using tertiles, missing values coded as missing), smoking (never, former, current), alcohol use (never, former, current), sedentary behavior (0-3h, 4-5h, 6-7h, >8h of daily TV watching, PC use during leisure, and driving), and screening for breast and/or bowel cancer (binary) as categorical variables, as well as body mass index (kg/m^2), and diet (healthy diet score, 0-7 scale) as continuous variables.

Supplementary Table 5. Hazard ratios and 95% confidence intervals for the proteomic signature and physical activity under single and mutual adjustment scenarios

**Outcome**	**Model adjustment**	**Signature**	**MVPA**
		Hazard ratio (95% CI)	Hazard ratio (95% CI)
Baseline to cancer	Single	0.84 (0.76, 0.92)	0.90 (0.84, 0.96)
	Mutual	0.87 (0.79, 0.96)	0.92 (0.86, 0.99)
Baseline to CVD	Single	0.97 (0.92, 1.02)	0.99 (0.96, 1.02)
	Mutual	0.97 (0.92, 1.03)	0.99 (0.96, 1.03)
Baseline to T2D	Single	0.67 (0.61, 0.73)	0.91 (0.86, 0.96)
	Mutual	0.67 (0.62, 0.74)	0.99 (0.93, 1.04)
Cancer to multimorbidity	Single	1.09 (0.76, 1.56)	0.97 (0.80, 1.17)
	Mutual	1.12 (0.77, 1.63)	0.95 (0.78, 1.16)
CVD to multimorbidity	Single	0.77 (0.59, 0.99)	0.93 (0.78, 1.11)
	Mutual	0.77 (0.59, 1.01)	0.99 (0.82, 1.18)
T2D to multimorbidity	Single	1.38 (0.90, 2.12)	1.00 (0.85, 1.17)
	Mutual	1.39 (0.90, 2.15)	0.99 (0.84, 1.16)

CI: confidence interval; CVD: cardiovascular disease; MVPA: moderate-to-vigorous physical activity; T2D: type 2 diabetes.

All Cox regression models were stratified by age at baseline (5-year increments), sex, and country (England, Scotland, Wales), and adjusted for education level (highest, intermediate, lowest, none of those), socio-economic status (Townsend index, categorized using tertiles, missing values coded as missing), smoking (never, former, current), alcohol use (never, former, current), sedentary behavior (0-3h, 4-5h, 6-7h, >8h of daily TV watching, PC use during leisure, and driving), and screening for breast and/or bowel cancer (binary) as categorical variables, as well as body mass index (kg/m^2), and diet (healthy diet score, 0-7 scale) as continuous variables.

Supplementary Figure 1. Flowchart of participant inclusion

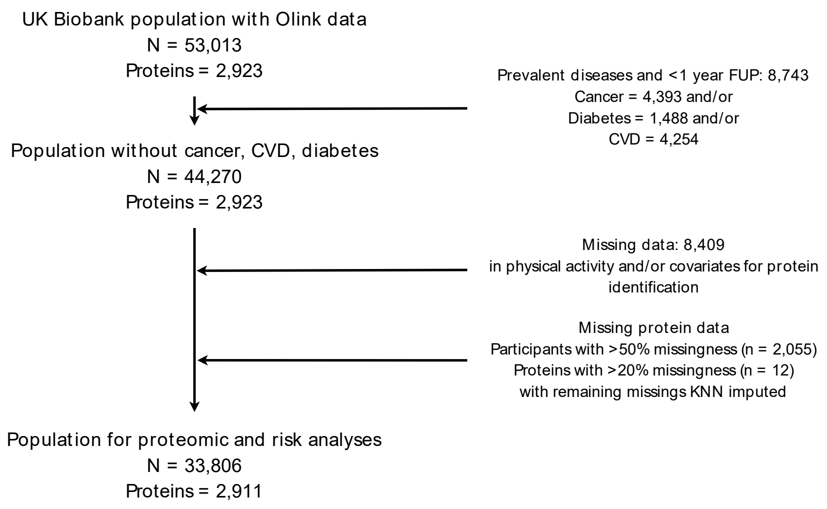

Legend: CVD: cardiovascular disease; FUP: follow-up; KNN: k-nearest-neighbor.

Supplementary Figure 2. Continuous hazard ratios for the top hits among all proteins for the disease-free to incident disease transitions

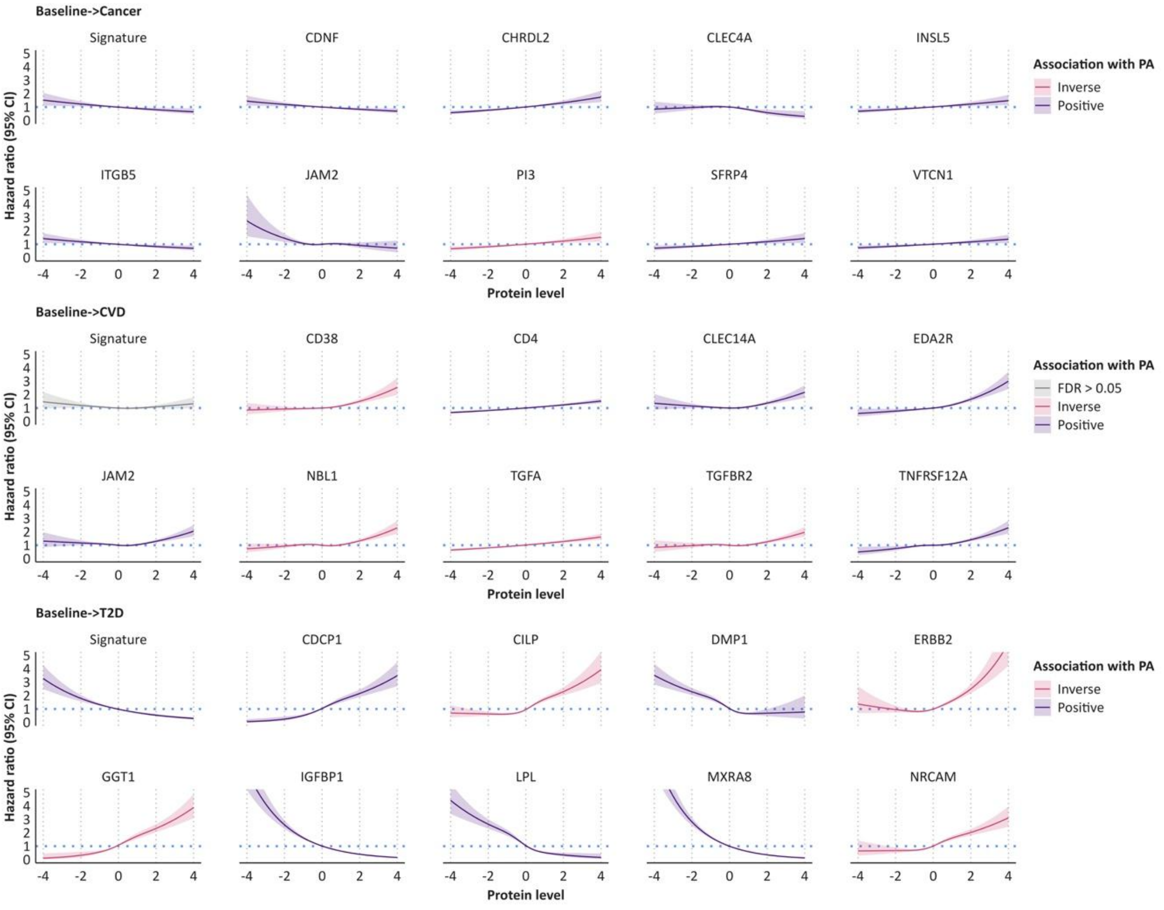

Legend: CI: confidence interval; CVD: cardiovascular disease; FDR: false discovery rate; T2D: type 2 diabetes; PA: physical activity.

Supplementary Figure 3. Continuous hazard ratios for the top hits among all proteins for the incident disease to subsequent multimorbidity transitions

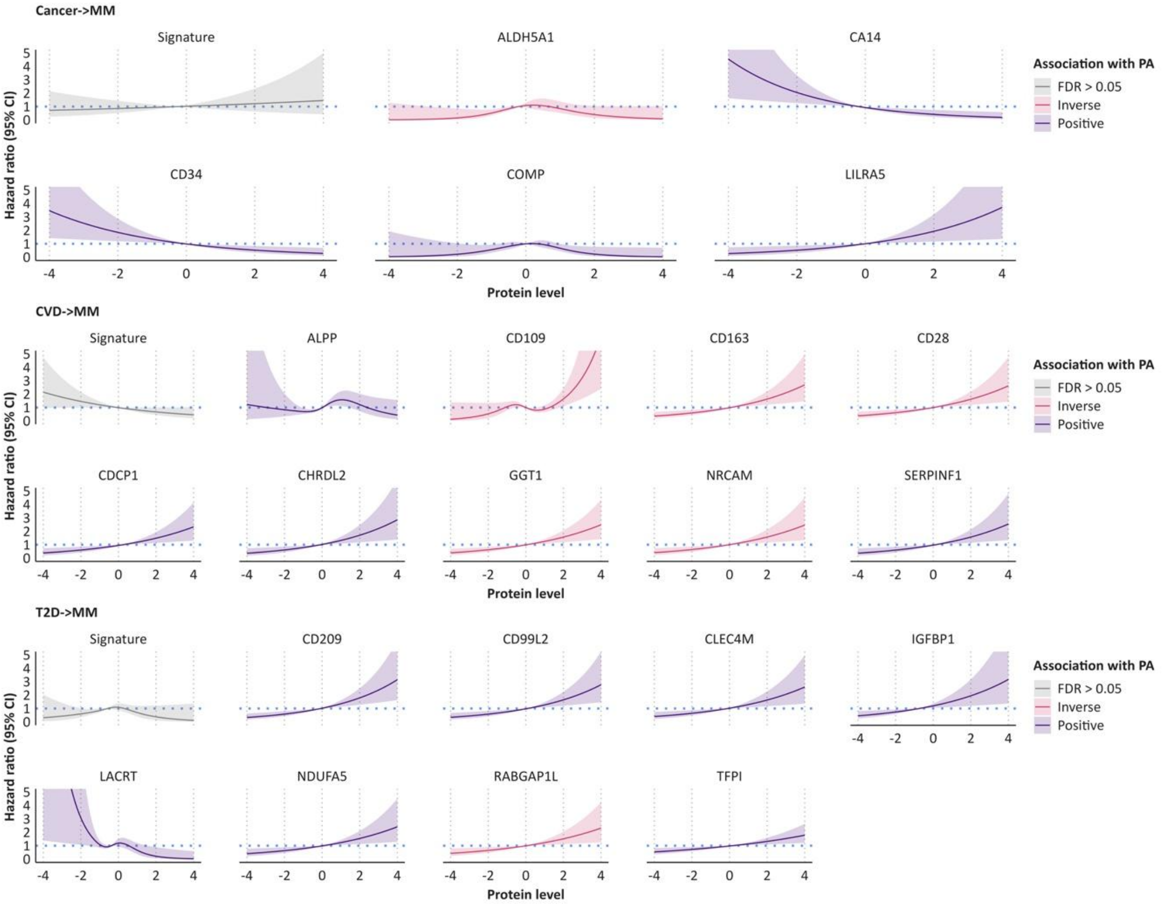

Legend: CI: confidence interval; CVD: cardiovascular disease; FDR: false discovery rate; MM: Multimorbidity; PA: physical activity; T2D: type 2 diabetes.
