# Supplementary material for: Proteomics signature of moderate-to-vigorous physical activity and risk of multimorbidity of cancer and cardiometabolic diseases
Source: Commun Med (Lond). 2026 Mar 13;6:160. doi: 10.1038/s43856-026-01514-9 (PMC13009281; doi:10.1038/s43856-026-01514-9)
Supplement: Supplementary file 3 — Description of Additional Supplementary files [file 43856_2026_1514_MOESM3_ESM.docx]

**Description of Additional Supplementary Files**

Supplementary Data 1: Regression coefficients with the full names of all identified proteins.

Supplementary Data 2: Source data for the figures 1B, 1C, 3, and 4
